# Supplementary material for: Structural basis of Spliced Leader RNA recognition by the Trypanosoma brucei cap-binding complex
Source: Nat Commun. 2025 Jan 15;16:685. doi: 10.1038/s41467-024-55373-w (PMC11735809; doi:10.1038/s41467-024-55373-w)
Supplement: Supplementary file 1 — Supplementary Information [file 41467_2024_55373_MOESM1_ESM.pdf]

Structural basis of Spliced Leader RNA recognition by the *Trypanosoma brucei* cap-binding complex

Harald Bernhard, Hana Petržílková, Barbora Popelářová, Kamil Ziemkiewicz, Karolina Bartosik, Marcin Warmiński, Laura Tengo, Henri Gröger, Luciano G. Dolce, Cameron D. Mackereth, Ronald Micura, Jacek Jemielity, and Eva Kowalinski*

* corresponding author email: kowalinski@embl.fr

S1

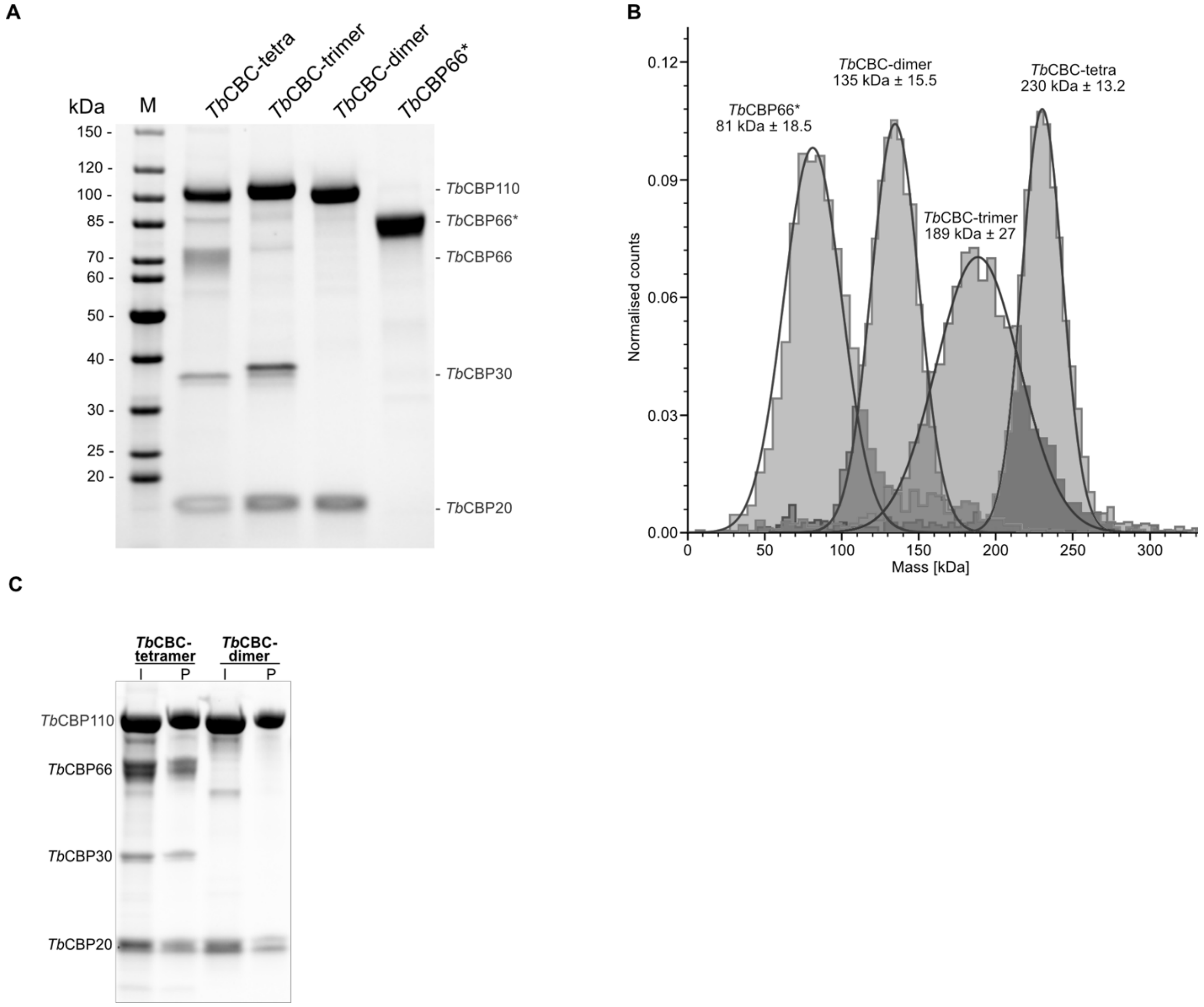

Figure S1: (A) Purity of *Tb*CBC complex samples. A Coomassie-stained 12% SDS-PAGE of the purified protein and protein complexes used in the study, representative gel of more than three replicates. **(B)** Mass photometry histograms of the samples. **(C)** Cap-binding assay on immobilized γ-Aminophenyl-m^7GTP resin with TbCBC-tetramer and TbCBC-dimer samples. I = input, P = pull-down. A Coomassie-stained 12 % SDS-PAGE is shown, representative gel of three repetitions of the assay. Source data are provided as a Source Data file.

S2

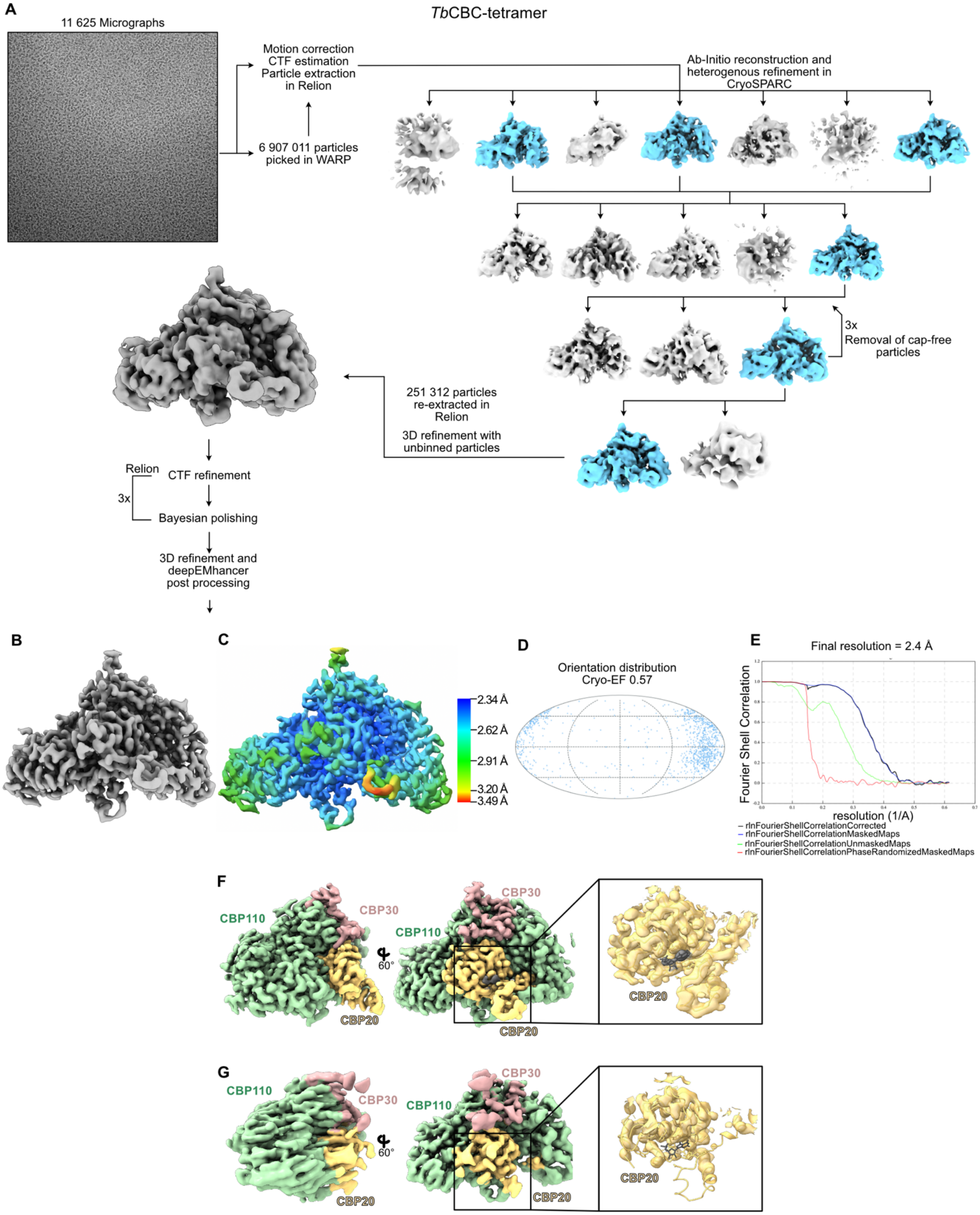
A
11 625 Micrographs
TbCBC-tetramer
Motion correction
CTF estimation
Particle extraction
in Relion
6 907 011 particles
picked in WARP
Ab-Initio reconstruction and
heterogenous refinement in
CryoSPARC
3x
Removal of cap-free
particles
251 312 particles
re-extracted in
Relion
3D refinement with
unbinned particles
Relion
CTF refinement
3x
Bayesian polishing
3D refinement and
deepEMhancer
post processing
B
C
2.34 Å
2.62 Å
2.91 Å
3.20 Å
3.49 Å
D
Orientation distribution
Cryo-EF 0.57
E
Final resolution = 2.4 Å
Fourier Shell Correlation
resolution (1/A)
rlnFourierShellCorrelationCorrected
rlnFourierShellCorrelationMaskedMaps
rlnFourierShellCorrelationUnmaskedMaps
rlnFourierShellCorrelationPhaseRandomizedMaskedMaps
F
CBP110
CBP30
CBP20
60°
G
CBP110
CBP30
CBP20
60°

Figure S2: EM processing of *Tb*CBC-tetramer. (A) Representative micrograph and schematic of the processing steps used. Particles from classes in blue were pooled for subsequent steps, and particles from classes in grey were discarded. **(B)** Final map from RELION 3D refinement post-processed by DeepEMhancer. **(C)** Local resolution. **(D)** Angular distribution map of particle orientation of the final refinement step in RELION. **(E)** Final resolution of the reconstruction, as calculated by RELION Gold Standard Fourier Shell Correlation, with a resolution of 2.4 Å when adopting a cutoff of 0.143. **(F)** Final cryo-EM map of *Tb*CBC-tetramer with focus on N-terminal domain. **(G)** Originally discarded particles (in grey) were sorted to calculate a ligand-free map. This apo-reconstruction contained 324 127 particles with a resolution of 3 Å and was highly anisotropic due to a preferred orientation of the particles on the grid. The same orientations as in (F) are shown to highlight the absence of the cap ligand and disorder of the N-terminal domain.

S3

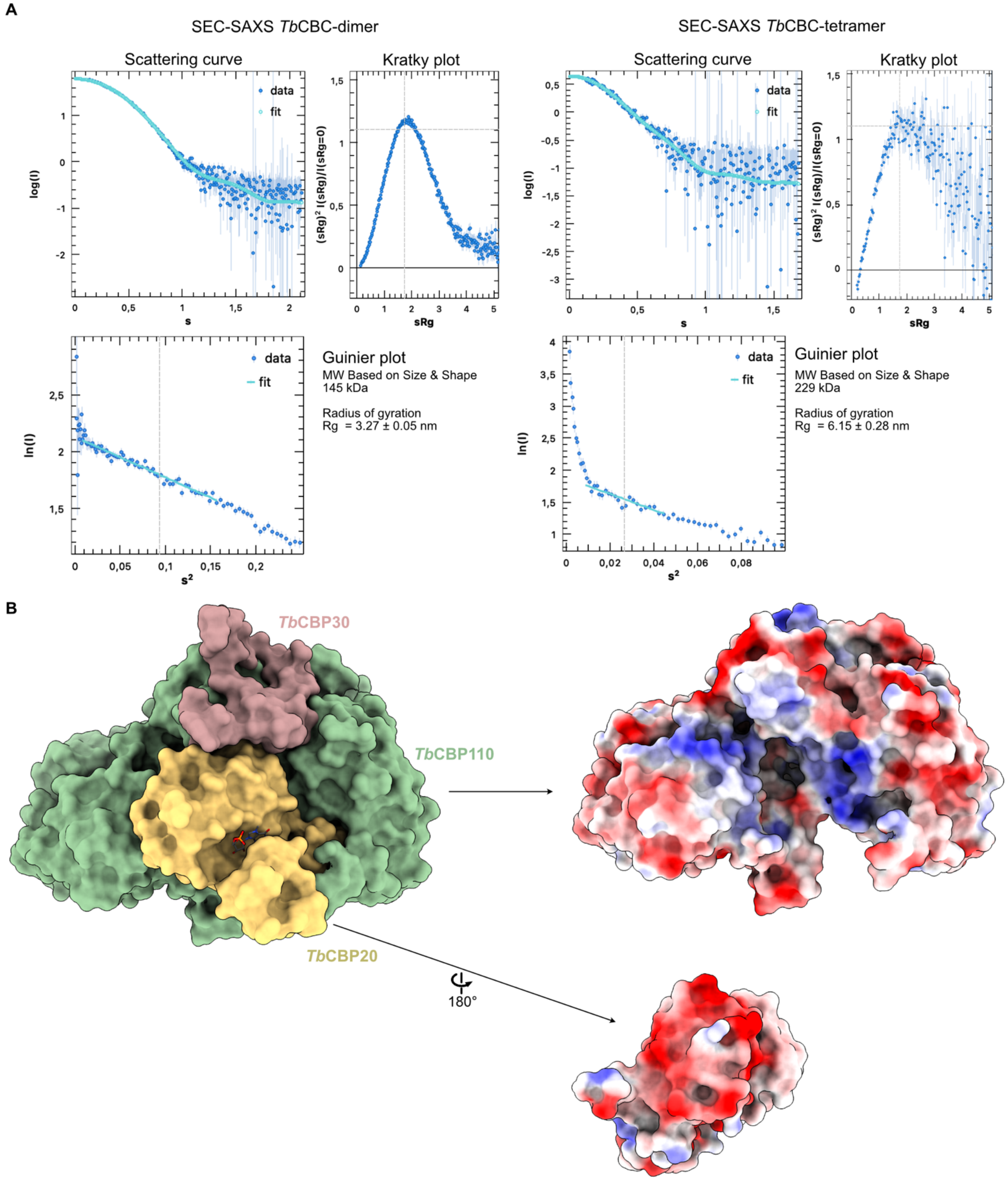
A
SEC-SAXS TbCBC-dimer
Scattering curve
Kratky plot
data
fit
log(I)
s
(sRg)² I(sRg)/I(sRg=0)
sRg
Guinier plot
MW Based on Size & Shape
145 kDa
Radius of gyration
Rg = 3.27 ± 0.05 nm
ln(I)
s²
SEC-SAXS TbCBC-tetramer
Scattering curve
Kratky plot
Guinier plot
MW Based on Size & Shape
229 kDa
Radius of gyration
Rg = 6.15 ± 0.28 nm
B
TbCBP30
TbCBP110
TbCBP20
180°

Figure S3: Architecture of *Tb*CBC. (A) Size-exclusion chromatography coupled small-angle X-ray scattering (SEC-SAXS) of *Tb*CBC dimer and tetramer. Scattering curves, normalized Kratky plots and Guinier plots of the TbCBC-tetramer sample in comparison with the TbCBC-dimer sample. The SEC-SAXS experiment was run once, the plots integrate the SEC peak frames. Error bars indicate the standard deviation from the mean of the scattering pattern. **(B)** The CBP20-CBP110 interface. Surface representation of *Tb*CBC-tetramer colored as in main Figure 1 (TbCBP110 green, *Tb*CBP20 yellow, *Tb*CBP30 pink) and surface representation of individual *Tb*CBP110 and *Tb*CBP20, colored by surface electrostatic charge as determined by ChimeraX's coulombic tool. Source data are provided as a Source Data file.

S4

A

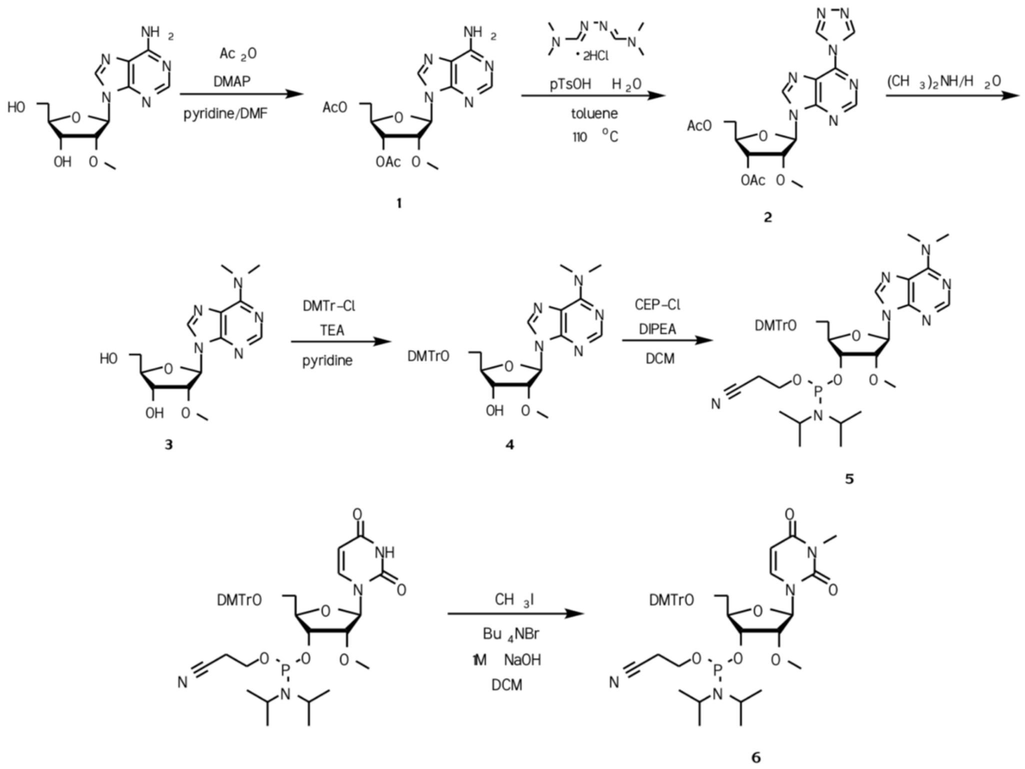
Ac2O
DMAP
pyridine/DMF
pTsOH H2O
toluene
110 °C
(CH3)2NH/H2O
DMTr-Cl
TEA
pyridine
CEP-Cl
DIPEA
DCM
CH3I
Bu4NBr
1M NaOH
DCM
1
2
3
4
5
6

B

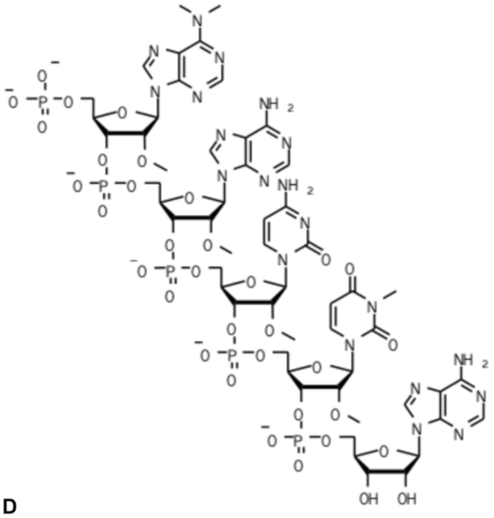

C

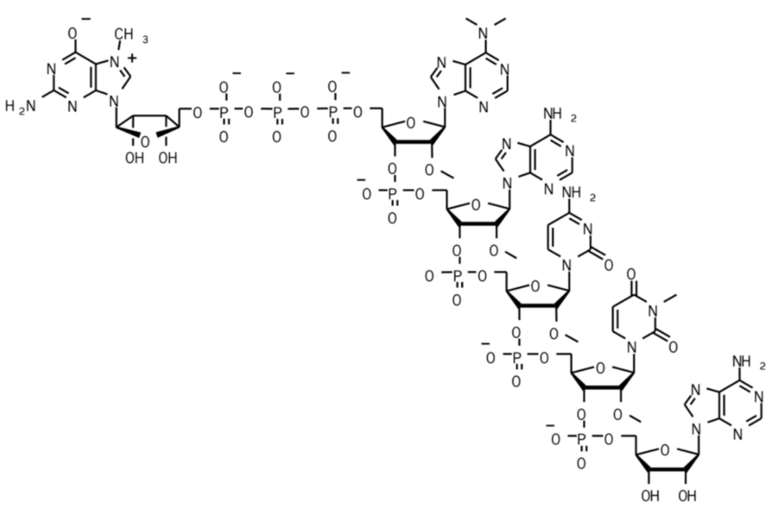

D

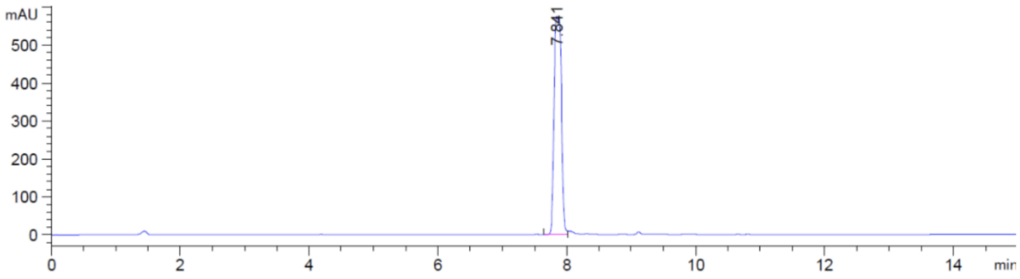
mAU
7.841
min

Figure S4: Synthesis of *T. brucei* cap4 ligand. (A, B, C) Schematics of the individual steps involved in the preparation of cap4. **(D)** Example HPLC profile of $m^7Gppp^{m6,6}A_mpA_mpCp^{m3}U_mpA$.

S5

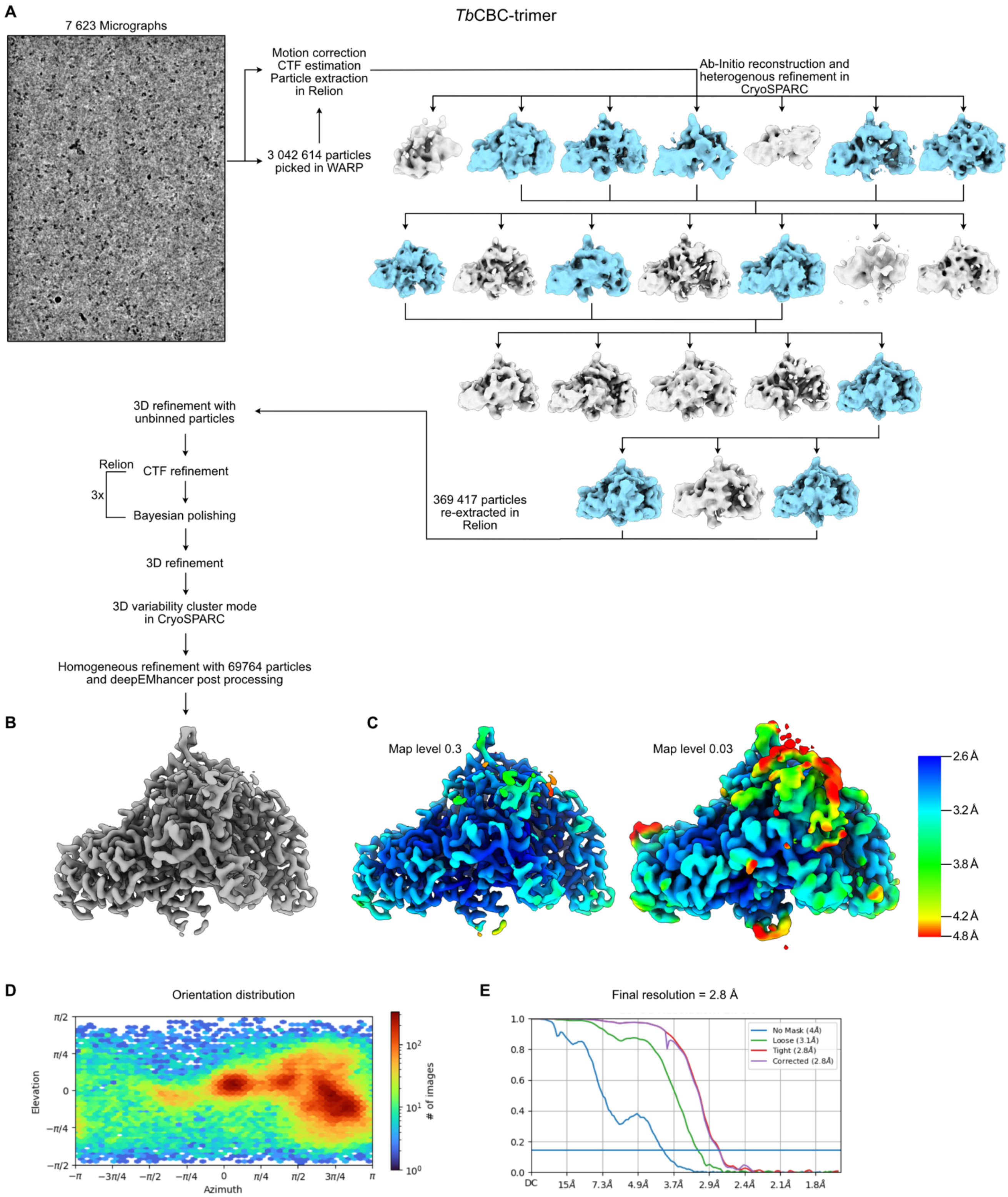
A
7 623 Micrographs
TbCBC-trimer
Motion correction
CTF estimation
Particle extraction
in Relion
3 042 614 particles
picked in WARP
Ab-Initio reconstruction and
heterogenous refinement in
CryoSPARC
3D refinement with
unbinned particles
Relion
CTF refinement
3x
Bayesian polishing
3D refinement
3D variability cluster mode
in CryoSPARC
Homogeneous refinement with 69764 particles
and deepEMhancer post processing
369 417 particles
re-extracted in
Relion
B
C
Map level 0.3
Map level 0.03
2.6 Å
3.2 Å
3.8 Å
4.2 Å
4.8 Å
D
Orientation distribution
Elevation
Azimuth
# of images
E
Final resolution = 2.8 Å
No Mask (4Å)
Loose (3.1Å)
Tight (2.8Å)
Corrected (2.8Å)
DC
15Å
7.3Å
4.9Å
3.7Å
2.9Å
2.4Å
2.1Å
1.8Å

Figure S5: EM processing of *Tb*CBC-trimer. (A) Representative micrograph and schematic of the processing steps used. Particles from classes in blue were pooled for subsequent steps, and particles from classes in grey were discarded. **(B)** Final map from cryosparc homogenous refinement and sharpened by DeepEMhancer. **(C)** Local resolution. **(D)** Angular distribution map of particle orientation of the final refinement step in CryoSPARC. **(E)** The final resolution of the reconstruction, as calculated by CryoSPARC Gold Standard Fourier Shell Correlation, with a resolution of 2.8 Å when adopting a cutoff of 0.143.

S6

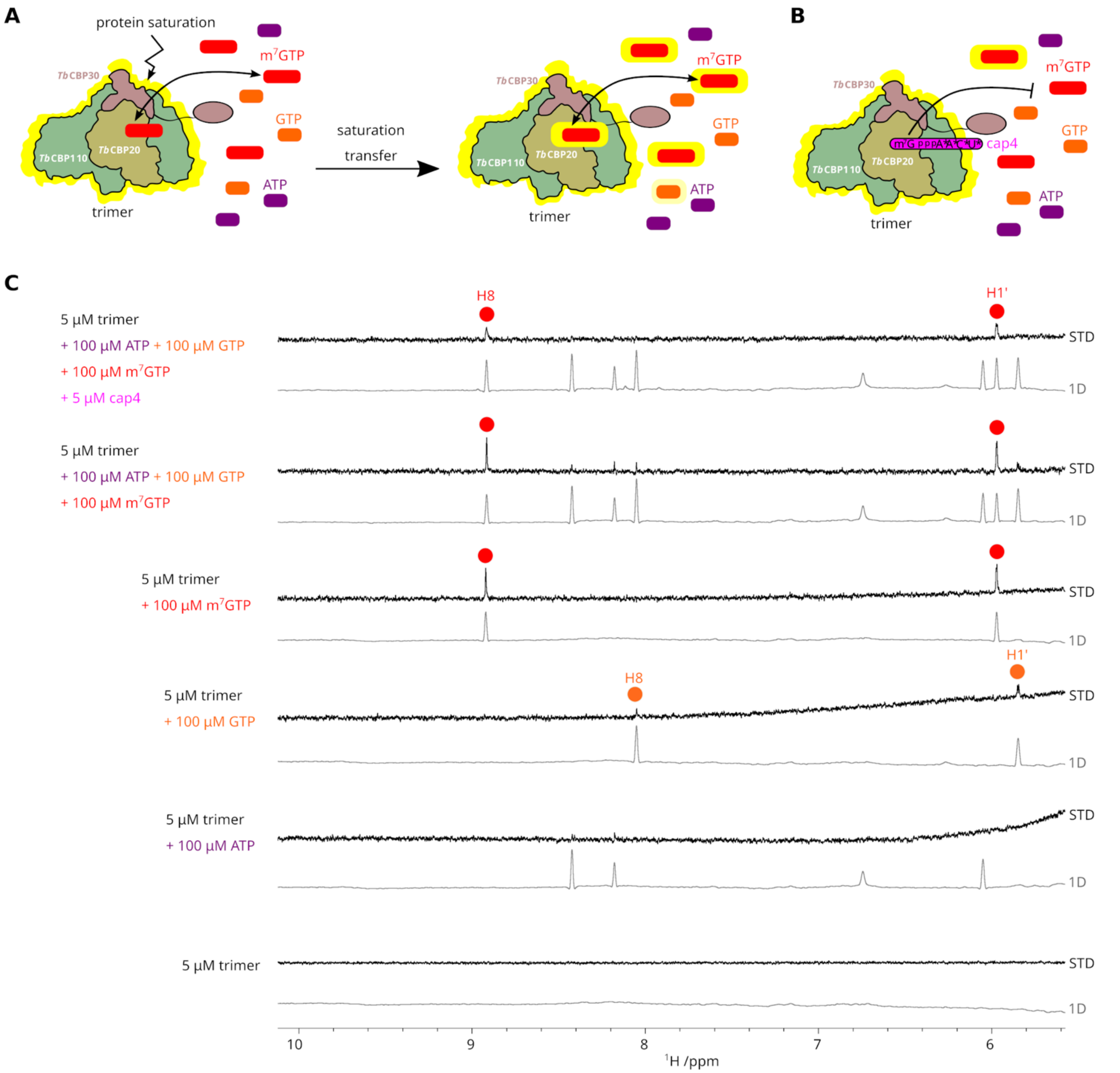

Figure S6: Nucleotide binding assayed by NMR spectroscopy. (A) The binding of ligands to the *Tb*CBC-trimer measured by using saturation transfer difference (STD). The protein (*Tb*CBC-trimer) is saturated at a ^{1}H frequency that matches signals from the protein but does not overlap with any resonance frequencies from the ligands (ATP, GTP, $m^{7}GTP$). As ligands bind and dissociate they acquire magnetization due to contact with the protein, and this transferred magnetization is visible by taking the difference between 1D ^{1}H spectra with and without protein saturation. Ligand peaks in the STD spectrum correspond to contacts with the protein (*Tb*CBC-trimer). The measurements can use a mixture of ligands (as shown) or with only one ligand. **(B)** The addition of cap4 RNA competes with binding by the triphosphate ligands. A reduction in the STD signal confirms that the triphosphate ligand(s) binds to the same site on the *Tb*CBC-trimer. **(C)** Series of STD measurements (STD) along with comparison 1D ^{1}H

NMR spectra (1D) of the samples. Starting from the bottom, the isolated 5 μM *Tb*CBC-trimer serves as a reference. Experiments in which only 100 μM ATP, 100 μM GTP or 100 μM m^7GTP have been added show that only m^7GTP has clear peaks in the STD spectrum (corresponding to the H8 and H1' nuclei), with only low-intensity signals for unmodified GTP and no peaks for ATP. In the presence of all three nucleoside triphosphates, the binding to m^7GTP is selective as it once again displays the highest signals in the STD spectrum. The further addition of the cap4 RNA to the sample corresponds with a decrease in the STD signals for m^7GTP, confirming that both ligands bind to the same location on the *Tb*CBC-trimer.

S7

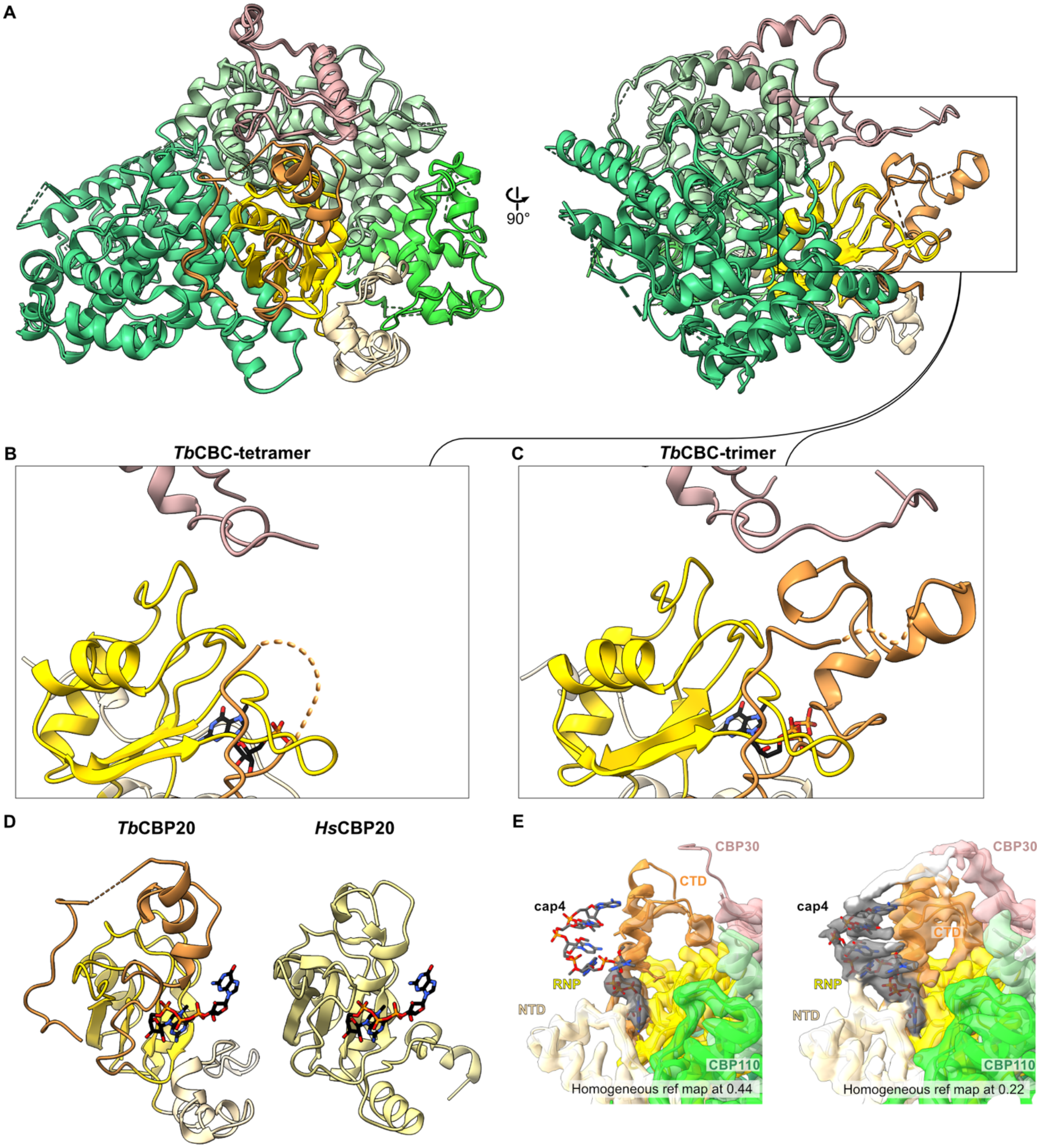

Figure S7: Comparison between *Tb*CBC-tetramer and *Tb*CBC-trimer models, and *T. brucei* and human CBP20. (A) Superimposition of *Tb*CBC-tetramer and *Tb*CBC-trimer in two orientations in cartoon representation. **(B)** Zoom into the differences between *Tb*CBC-tetramer and *Tb*CBC-trimer models comprising the C-terminal domain of *Tb*CBP20 and regions of *Tb*CBP30: *Tb*CBC-tetramer in **(B)** and *Tb*CBC-trimer in **(C). (D)** Side-by-side comparison of *Tb*CBP20 and *Hs*CBP20 (PDB:1H2T). Colors as in main Figure 1 and 3 (for *Tb*CBP20 domains). **(E)** CryoSPARC final homogeneous refinement trimer cryo-EM map transparent coloured as Figura 3 with cartoon model and m^7GTP in stick representation and at level 0.44 (left) and at a very reduced level of 0.22 (right). Unassigned density at the reduced level map in light gray.

S8

A

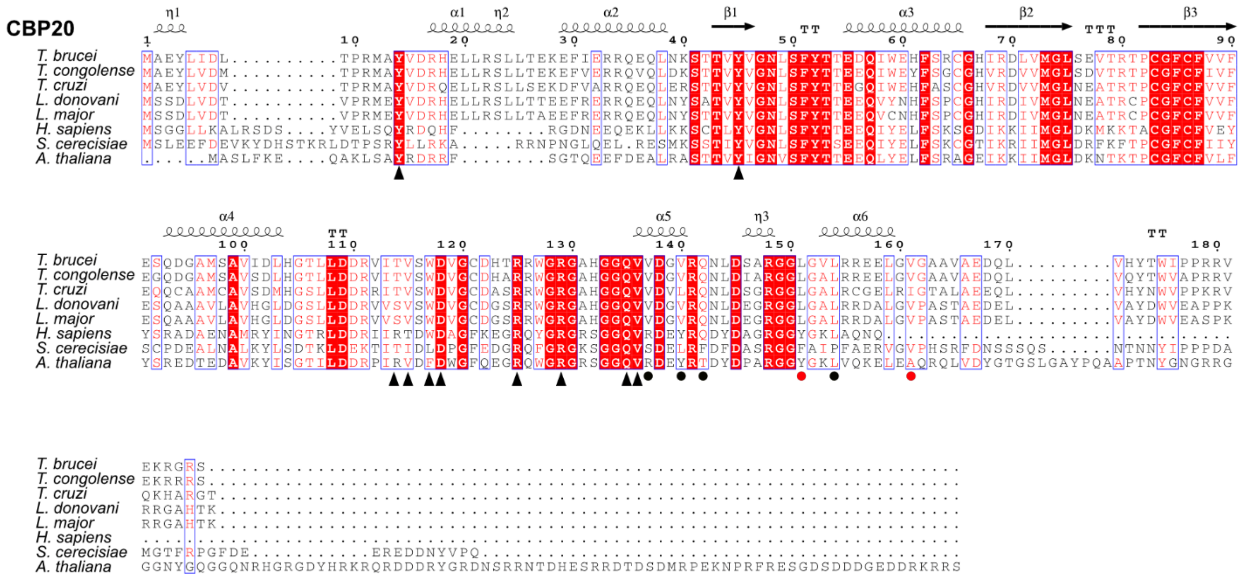
CBP20
T. brucei
T. congolense
T. cruzi
L. donovani
L. major
H. sapiens
S. cerecisiae
A. thaliana

B

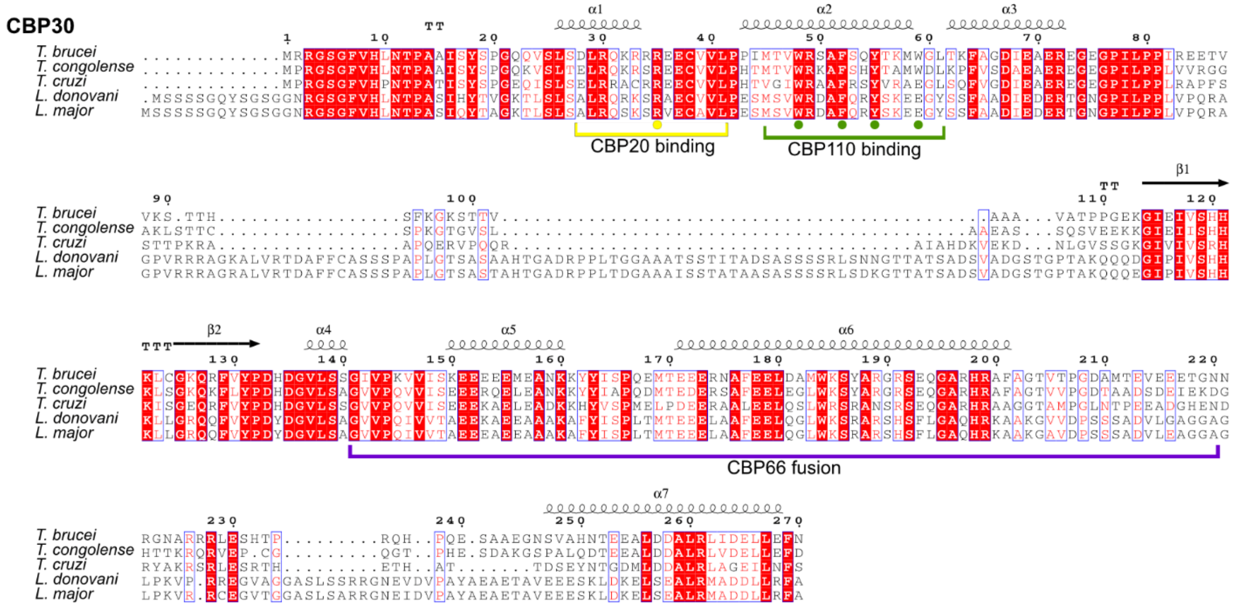
CBP30
T. brucei
T. congolense
T. cruzi
L. donovani
L. major
CBP20 binding
CBP110 binding
CBP66 fusion

S8 - continued

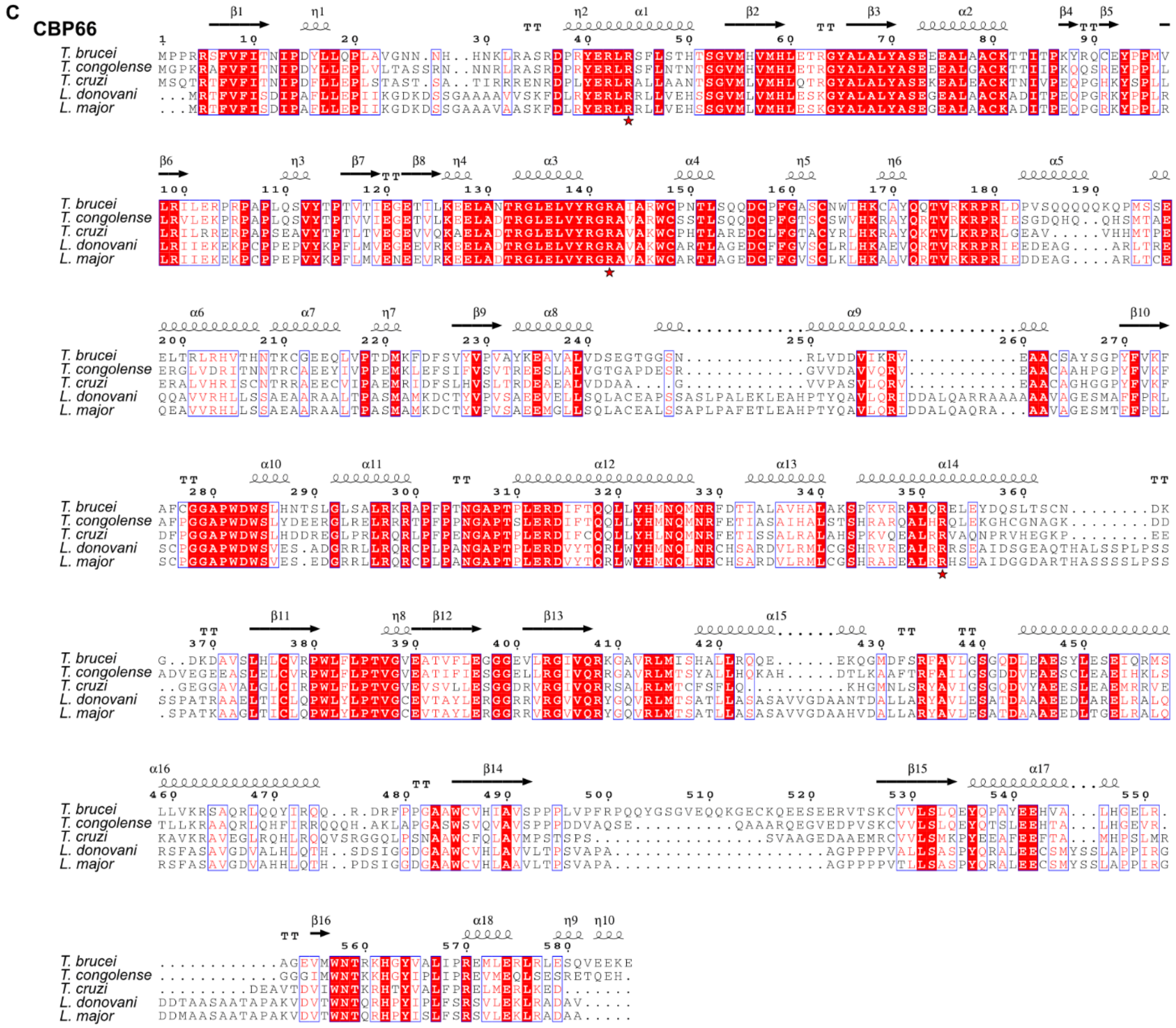

S8 – continued

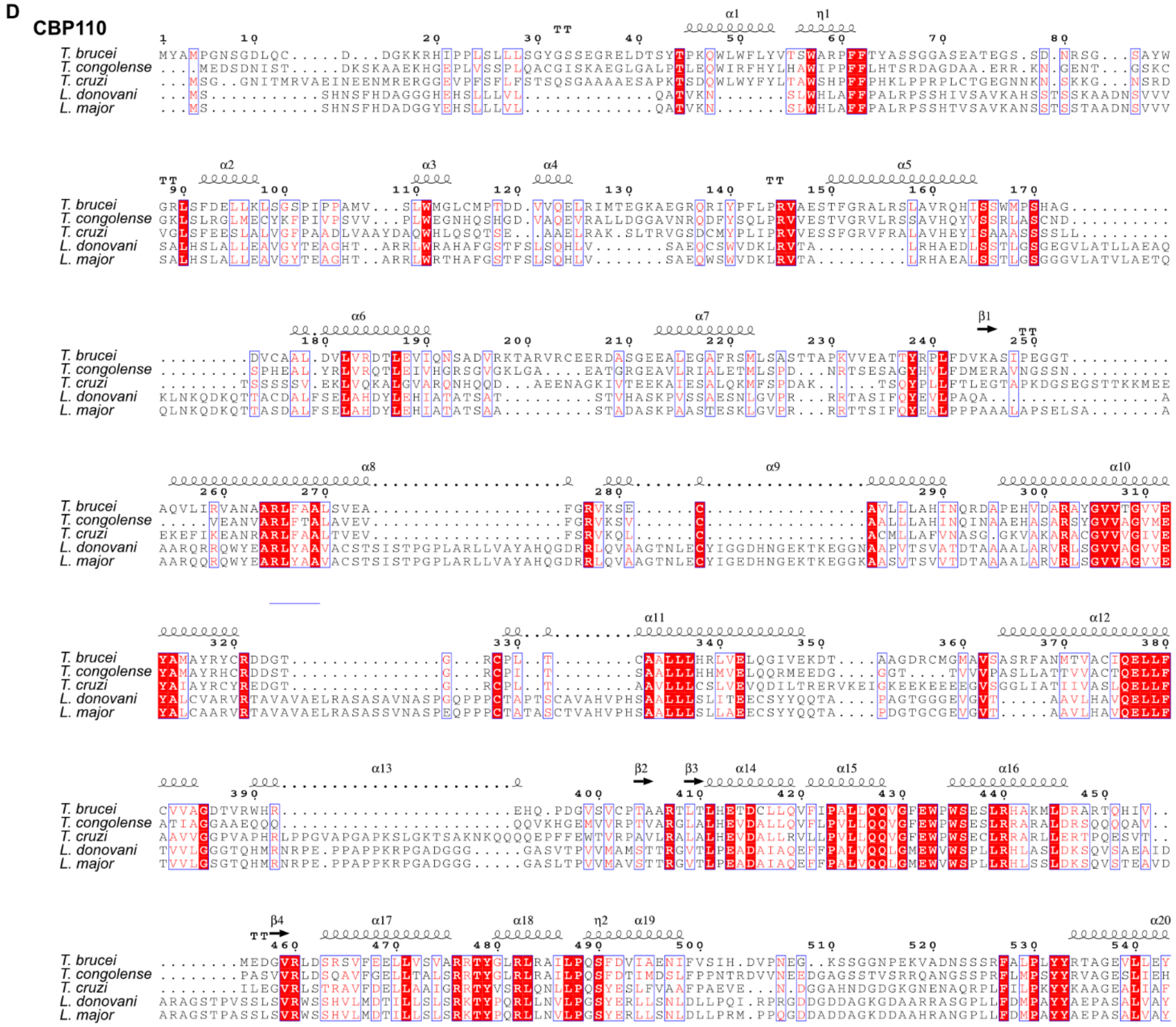

S8 – continued

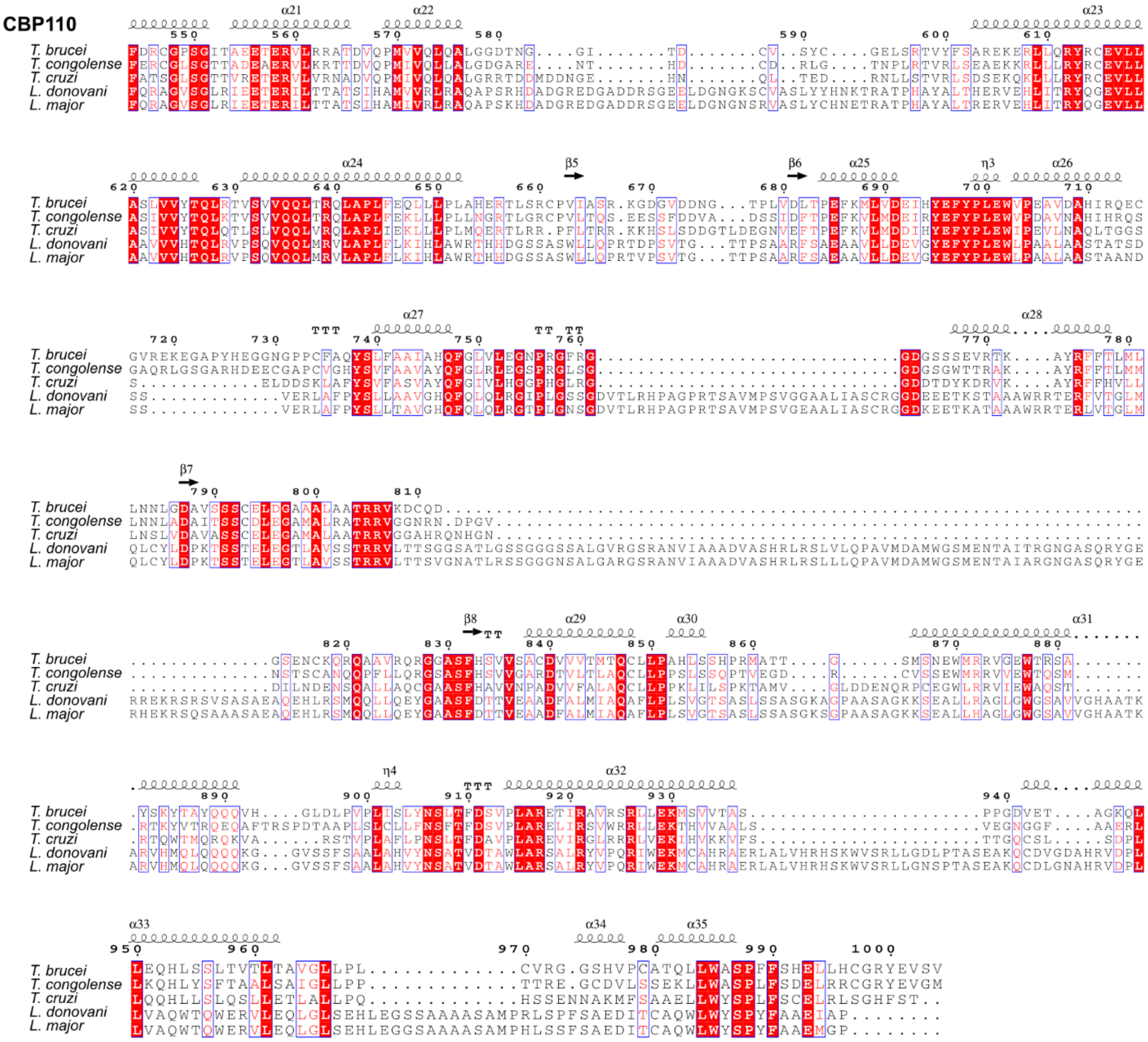

Figure S8: Sequence alignments of *Tb*CBC subunits. (A) Alignment of *Tb*CBP20 with the CBP20 of other trypanosomatids and to CBP20 of selected Opisthokonts. Black triangles highlight residues involved in cap binding. Black circles highlight kinetoplast-conserved residues involved in the interaction with cap4. Red circles highlight kinetoplast-conserved residues involved in the interaction with *Tb*CBP30 **(B)** Alignment of *Tb*CBP30 with the CBP30 of other trypanosomatids. Regions involved with interaction with *Tb*CBP20 and *Tb*CBP110 are highlighted in yellow and green, respectively, with residues mutated in this study highlighted with circles. The region used for the *Tb*CBP66* fusion construct is highlighted in purple. **(C)** Alignment of *Tb*CBP66 with the CBP66 of other trypanosomatids. Residues mutated in this study are highlighted with a red star. **(D)** Alignment of *Tb*CBP110 with the CBP110 of other trypanosomatids. Secondary structure annotations based on our structure for CBP20 and CBP110 and on AlphaFold DB prediction for CBP30 and CBP66

S9

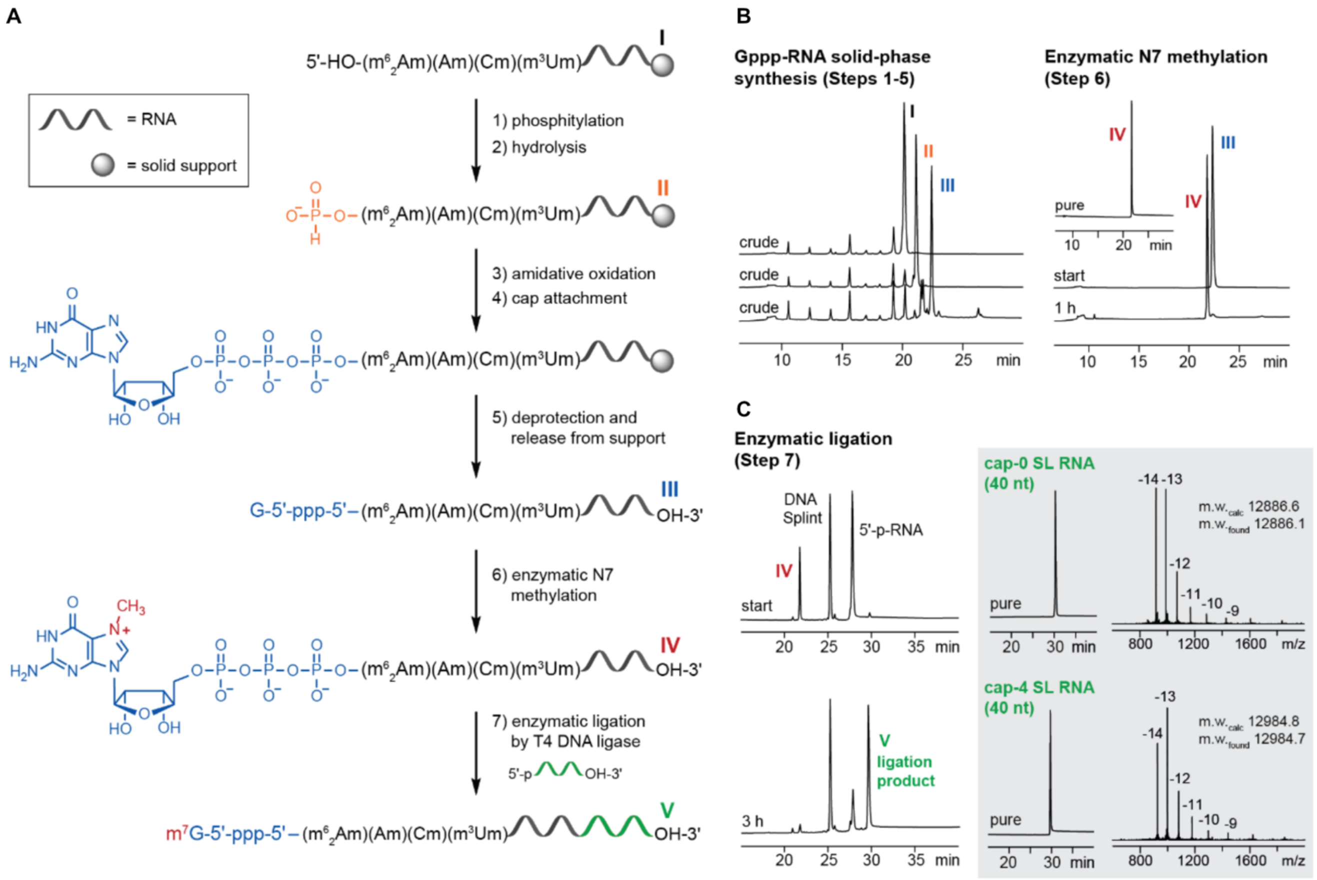

Figure S9: Synthesis of *T.brucei* cap0 SL RNA (40 nt) and cap4 SL RNA (40 nt). (A) Schematics of the individual steps involved in the preparation of cap4 RNA. The cap0 analog was prepared following the same procedure. **(B)** Left traces: Solid phase synthesis of short Gppp-RNA (steps 1-5). Reaction control of the individual steps was based on small portions of support-bound RNAs (I to III) that were individually deprotected and analyzed by AE HPLC. Right traces: Guanine N7 methylation of Gppp-RNA (step 6). HPLC profiles show the methylation reaction at the start (HPLC-purified oligomer III) and after 1 h; inset shows purified product of methylation (IV). **(C)** Example of enzymatic ligation of cap4 RNA (18 nt) and chemically synthesized 5'-p RNA (22 nt) using T4 DNA ligase and DNA splint (23 nt). The left HPLC profiles show sample composition at the start and after completion of the reaction (3 h). The middle traces show the HPLC-purified ligation products (V) that were further characterized by mass spectrometry (right traces).

S10

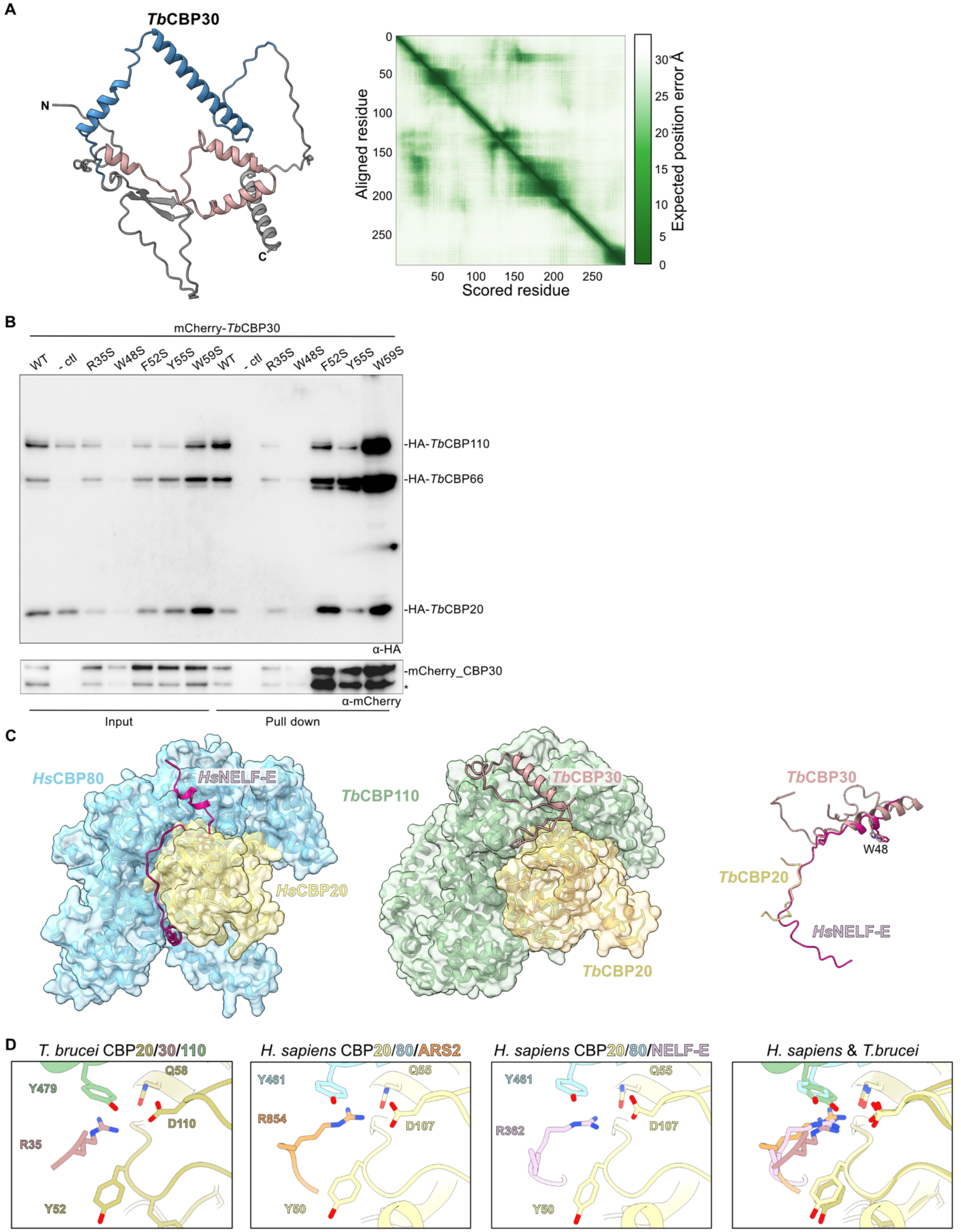
A
TbCBP30
N
C
Aligned residue
Scored residue
Expected position error Å
B
mCherry-TbCBP30
WT
- ctl
R35S
W48S
F52S
Y55S
W59S
-HA-TbCBP110
-HA-TbCBP66
-HA-TbCBP20
α-HA
-mCherry_CBP30
*
α-mCherry
Input
Pull down
C
HsCBP80
HsNELF-E
HsCBP20
TbCBP110
TbCBP30
TbCBP20
W48
D
T. brucei CBP20/30/110
H. sapiens CBP20/80/ARS2
H. sapiens CBP20/80/NELF-E
H. sapiens & T.brucei
Y479
Q58
R35
D110
Y52
Y461
Q55
R854
D107
Y50
R362

Figure S10: *Tb*CBC30 interactions. (A) AlphaFold2 model of *Tb*CBP30 and corresponding predicted aligned error (PAE) plot. The *Tb*CBP20 interaction region is colored in pink, and the helical region used to create the *Tb*CBP66* fusion construct is colored in blue. **(B)** Co-immunoprecipitation assay of co-expressed *Tb*CBP20, *Tb*CBP110, *Tb*CBP66 with mCherry-*Tb*CBP30 mutants and Western blot detection (compare to main Figure 4C). **(C)** Comparison of the NELF-E binding on *human* CBP20-CBP80 dimer (left) with the *Tb*CBP30 peptide binding on *Tb*CBP20-*Tb*CBP110 dimer (middle). Right panel: only the binding peptides are shown. **(D)** Comparison of binding of arginine R35 (*Tb*CBP30) with the binding or arginines in the same position of the human co-factors ARS2 and NELF-E on the human CBC (PDB: 5OO6 and 5OOB). Source data are provided as a Source Data file.

S11

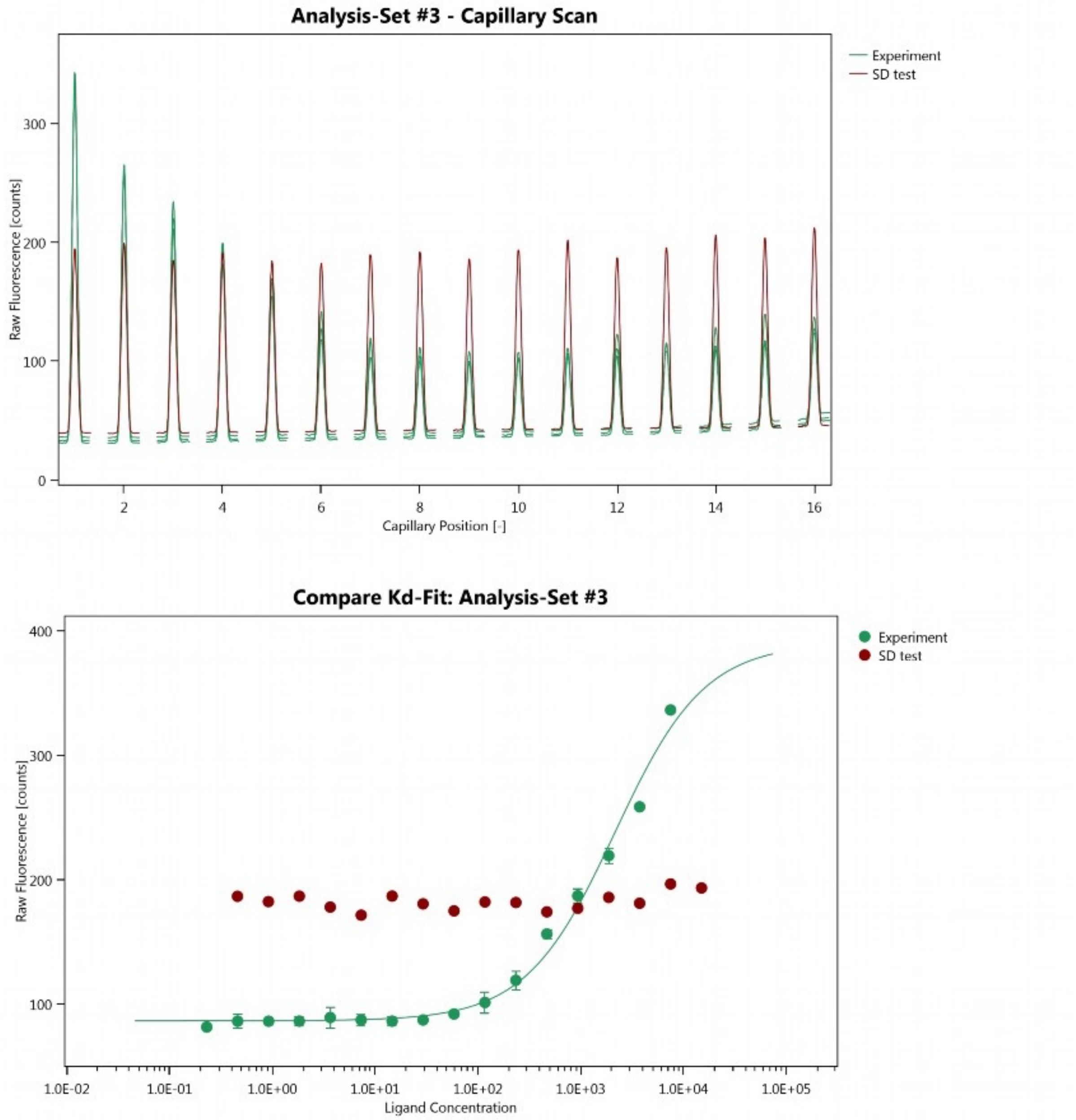

Figure S11: Microscale thermophoresis SDS-denaturation (SD) test, used to validate the approach of initial fluorescence variation for the dissociation constant estimation. The top panel represents the capillary scan, and the bottom represents the plotted data, with an experimental run in green and the SD test in red. SD test n=1; measurement n=3 with. Error bars indicate standard deviation from mean .Source data are provided as a Source Data file.

S12

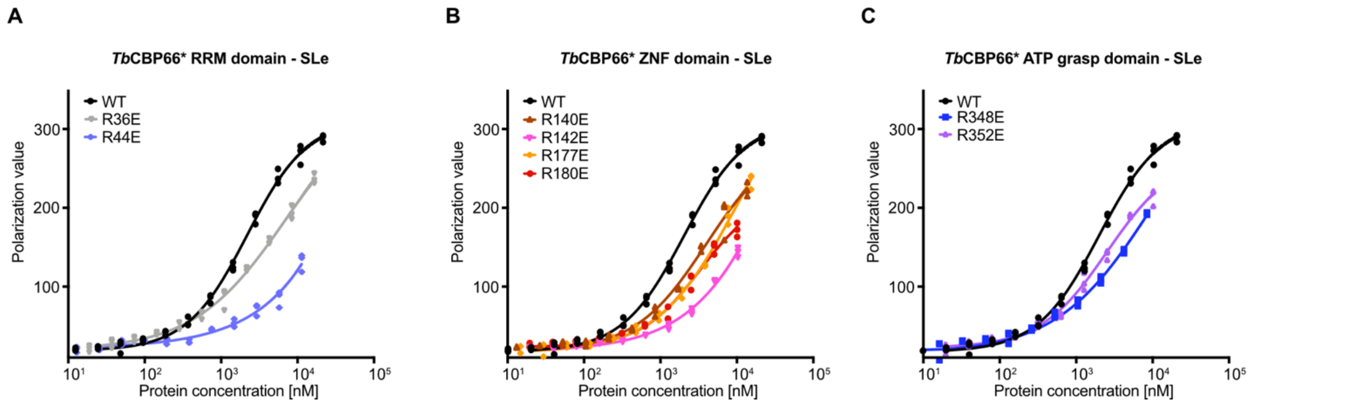

Figure S12: Fluorescence polarization (FP) assay probing the binding ability of *Tb*CBP66* mutants to the 39 nt SL RNA exon. **(A)** Mutants in the RRM domain. **(B)** Mutants in the ZNF domain. **(C)** Mutants in the ATP grasp domain. Measurements were run in triplicate and fit with a nonlinear fit model (n=3). Source data are provided as a Source Data file.

Supplementary Table S1: Theoretical mass and measured molecular weight, determined by mass photometry of all *Tb*CBCs.

Protein complex	Theoretical MW	Measured MW	Events detected
*Tb*CBC-tetramer	228 kDa	230 kDa ± 13.2 kDa	1345
*Tb*CBC-trimer	162 kDa	189 kDa ± 27 kDa	2283
*Tb*CBC dimer	132 kDa	135 kDa ± 15.5 kDa	3066
*Tb*CBC66*	76 kDa	81 kDa ± 18.5 kDa	1684

Supplementary Table S2: Affinity of different protein complexes and RNA as determined by RNA-EMSA

Protein complex	RNA	k_D (nM)	sdev	Hill factor
*Tb*CBC-dimer	cap4-SLe	620	46 - 1376	0.59
*Tb*CBC-dimer	cap0-SLe	668	395 - 996	0.81
*Tb*CBC-dimer	OH-SLe	N/A	N/A	0.88
*Tb*CBC-trimer	cap4-SLe	208	102 - 320	0.77
*Tb*CBC-trimer	cap0-SLe	179	89 - 272	0.84
*Tb*CBC-trimer	OH-SLe	3805	2838 - 5548	1.76
*Tb*CBC-tetramer	cap4-SLe	47	20 - 67	1.42
*Tb*CBC-tetramer	cap0-SLe	39	9 - 61	1.27
*Tb*CBC-tetramer	OH-SLe	135	82 - 193	1.06
*Tb*CB66*	OH-SLe	618	494 - 760	1.19
*Tb*CB66*	15nt-single stranded	N/A	N/A	N/A
*Tb*CB66*	SL-hairpin_loop	390.0	296 - 502	2.01

Supplementary Table S3: Affinity of *Tb*CBP66 and OH-SLe RNA as determined by fluorescence polarization

Protein complex	RNA	k_D (nM)	sdev	Hill factor
*Tb*CB66* WT	OH-SLe	2000	1801 - 2253	1.07
*Tb*CB66* R352E	OH-SLe	2571	1955 - 4099	0.99
*Tb*CB66* R142E	OH-SLe	N/A	N/A	N/A
*Tb*CB66* R44E	OH-SLe	N/A	N/A	N/A
*Tb*CB66* R36E	OH-SLe	6949	4055 - 22449	0.69
*Tb*CB66* R140E	OH-SLe	3905	2704 - 7705	0.93
*Tb*CB66* R177E	OH-SLe	10090	5751 - 33720	0.82
*Tb*CB66* R180E	OH-SLe	3199	2386 - 5500	1.12
*Tb*CB66* R348E	OH-SLe	9808	N/A	0.69

Supplementary Table S4: Gene accession number of *Tb*CBC subunits.

Protein name	Gene accession number	UniProt ID	Tag
*Tb*CBP20	Tb927.6.1970	Q585L4	None
*Tb*CBP30	Tb927.10.15210<br>Tb10.61.1300	Q387Z0	None
*Tb*CBP66	Tb927.3.1340	Q57XW7	None
*Tb*CBP110	Tb927.10.2990<br>Tb10.70.4560	Q38BU6	N-Terminal 8xHis

Supplementary Table S5. Sequences and characterization data of chemically and chemo-enzymatically synthesized RNAs.

RNA	Sequence 5' to 3'	nt	Scale	Isolated yield*	m.w. (calc.)	m.w. (found)
cap0	Gppp-AACUAACGCUAUUAUUA	18	1 µmol	42 nmol	5845.4	5845.1
	m^7Gppp-AACUAACGCUAUUAUUA	18	30 nmol	22 nmol	5859.5	5859.2
	m^7Gppp-AACUAACGCUAUUAUUA-GAACAGUUUCUGUACUAUAUUG	40	10 nmol	6 nmol	12886.6	12886.1
cap4	Gppp-(m^6_2Am)(Am)(Cm)(m^3Um)AA-CGCUAUUAUUA	18	1 µmol	34 nmol	5943.6	5943.7
	m^7Gppp-(m^6_2Am)(Am)(Cm)(m^3Um)-AACGCUAUUAUUA	18	30 nmol	20 nmol	5957.7	5957.6
	m^7Gppp-(m^6_2Am)(Am)(Cm)(m^3Um)-AACGCUAUUAUUAGAACAGUUU-CUGUACUAUAUUG	40	10 nmol	5 nmol	12984.8	12985.0

*Amount of pure product obtained after HPLC purification
